# *Plasmodium yoelii* 17XL infection modified maturation and function of dendritic cells by skewing Tregs and amplificating Th17

**DOI:** 10.1186/s12879-020-04990-z

**Published:** 2020-04-06

**Authors:** Guang Chen, Ji-wei Du, Qing Nie, Yun-ting Du, Shuang-chun Liu, De-hui Liu, Hui-ming Zhang, Fang-fang Wang

**Affiliations:** 1 Department of Basic Medical Sciences, Taizhou University Hospital, Taizhou University, No 1139 Shifu Road, Jiaojiang District, Taizhou, 318000 China; 2 Nursing Department, Xiang’An Hospital, Xiamen University, No 2000, Xian’an East Road, Xiang’an District, Xiamen, 361005 China; 3 Weifang Centers for Disease Control and Prevention, No 4801 Huixian Road, Gaoxin District, Shandong Province, Weifang, 261061 China; 4 Department of Laboratory Medicine, Cancer Hospital of China Medical University, Liaoning Cancer Hospital & Institute, No 44, Xiaoheyan Road, Dadong District, Shenyang, 110042 China; 5 Municipal Hospital Affiliated to Medical School of Taizhou University, No 381, Zhongshan East Road, Jiaojiang District, Taizhou, 318000 China; 6 College of Basic Medical Sciences, Jiamusi University, No 148 Xuefu Street, Jiamusi, 154007 China

**Keywords:** *Plasmodium yoelii* 17XL, Dendritic cells, Tregs, Th17, Malaria infection

## Abstract

**Background:**

Emerging data has suggested that Tregs, Th17, Th1 and Th2 are correlated with early immune mechanisms by controlling *Plasmodium* infection. *Plasmodium* infection appeared to impair the antigen presentation and maturation of DCs, leading to attenuation of specific cellular immune response ultimately. Hence, in this study, we aim to evaluate the relevance between DCs and Tregs/Th17 populations in the process and outcomes of infection with *Plasmodium yoelii* 17XL *(P.y*17XL).

**Methods:**

DCs detection/analysis dynamically was performed by Tregs depletion or Th17 neutralization in *P.y*17XL infected BALB/c mice via flow cytometry. Then the levels of cytokines production were detected using enzyme-linked mmunosorbent assay (ELISA).

**Results:**

Our results indicated that Tregs depletion or Th17 neutralization in BALB/c mice infected with *P.*y17XL significantly up-regulated the percentages of mDC and pDC, increased the expressions of major histocompatibility complex (MHC) class II, CD80, CD86 on DCs and the levels of IL-10/IL-12 secreted by DCs, indicating that abnormal amplification of Tregs or Th17 may damage the maturation and function of DCs during the early stage of malaria infection. Interestingly, we also found that the abnormal amplification of Th17, as well as Tregs, could inhibit the maturation of DCs.

**Conclusions:**

Tregs skewing or Th17 amplification during the early stage of malaria infection may inhibit the maturation and function of DCs by modifying the subsets of DCs, expressions of surface molecules on DCs and secretion mode of cytokines.

## Background

Malaria is attracting global attention as one of the world’s major infectious diseases due to its high morbidity and mortality. The World Health Organization (WHO) reported there were more than 200 million cases of malaria in 87 countries globally, which leads to approximately 435,000 deaths [1]. To be mentioned, the most vulnerable group affected by malaria are children under 5 year’s old and accounted for 61% (266000) of all malaria deaths worldwide [1]. Now it is most difficult to eradicate malaria due to lack of an efficacious antimalaria vaccine, the emergence of insecticide resistance in *Anopheles* mosquito vectors and increasing drug-resistant parasites [2, 3]. Thus, it is an urgent need to control the transmission of malaria infection, treat the patients infected by *plasmodium* and reduce the mortality. Beyond all questions, a better understanding of the underlying pathology and immunopathology of malaria is required.

Upon infection, immunity against parasite infecgtion plays a central role in clearing the parasite in the bloodstream. Innate immunity is firstly activated, e.g. complement system, innate lymphoid cells and dendritic cells (DCs), and then be in active intimate contact with parasite in blood but not enough to completely remove the infection [4–6]. Accumulating evidence indicated that CD4^+^ T cells have an essential role in the control of blood-stage malaria infection. Moreover, a new population of effector CD4^+^ helper T cells, identified as Th17, characterized by the secretion of IL-17, has some distinctions in phenotype, function and development compared with Th1 and Th2 cells [7, 8]. In addition, regulatory T cells (Tregs) is another distinct subset of CD4^+^ T cells, which has been clarified to downregulate immune responses through inhibition of effector cells [9]. Therefore, two cell subsets including Tregs and Th17, have the opposite effects in immune response.

DCs, as dedicated antigen presenting cells (APC), have vital roles in immune system during malaria infection. DCs are particularly important to take part in activation of CD4^+^ T cells, resulting in fighting against the parasites by producing inflammatory cytokines and activating other immune cells. Relative studies demonstrated that *Plasmodium* infection impaired the function of antigen presentation and the maturation of DCs in host [10–13]. Plasmacytoid and immature DCs can produce higher level of IL-10 in *P. yoelii*-infected mice. Furthermore, the expression of FoxP3, IL-17, transforming growth factor beta (TGF-β) and IL-6 were also different during *plasmodium* infection in different species [14]. Taken together, these discoveries indicated that CD4^+^ T cell subsets (Tregs, Th17) participate in the control of DCs function. Certain phenotypic and functional subsets of splenic DCs are the key regulators for modified pattern of immune responses to *P. yoelii* infections.

Rodent malaria parasite provides an available model for investigating the related mechanisms of human *plasmodium* infection. In this study we analyzed the phenotype and function characteristics of DCs proliferation, especially by rodent model with Tregs depletion or Th17 neutralization, to evaluate the relevance between DCs and Tregs/Th17 population in the process and outcomes of infection with *Plasmodium yoelii* 17XL *(P.y*17XL).

## Methods

### Mice, parasites, and experimental infection

BALB/c mice, female, 6–8 weeks old (weighted 18-25 g;) were purchased from the Beijing Animal Institute. Dr. Motomi Torii, from Department of Molecular Parasitology, Ehime University Graduate School of Medicine, Ehime, Japan, provided the *P.y*17XL. The animals were fed according experimental conditions with a 12 h light-dark cycle, a temperature of 22–24 °C and a humidity of 50 ± 5%. BALB/c mice were injected 1 × 10^6^*P.y*17XL parasitized erythrocytes by intraperitoneal (ip). and we monitored the dynamics of parasitaemia, which a light microscope was used to examine Giemsa-stained, thin (tail) blood smears. Mortality was monitored daily. According the Regulations for the Administration of Affairs Concerning Experimental Animals (1988.11.1), all animal were executed and humanely treated. The Institutional Animal Care and Use Committee (IACUC) of Taizhou University approved the protocols of the use of laboratory animals (Permit Number: 2007000542390). We didn’t submitted experimental animals to euthanasia during the process of *plasmodium* infection, which all mice died in day 4–8 p.i..

### CD25 depletion in *P.y*17XL-infected BALB/c mice

We used Anti-mouse CD25 mAb (7D4) to deplete Tregs. 1 mg 7D4 was administrated to BALB/c mice by i.p one day before and after parasite challenge. The same volume of phosphate-buffered saline (PBS) was injected by i.p in control group. Flow cytometry have detected the efficacy of Tregs depletion [15]. And we used spleen cell culture supernatants to analyze the levels of cytokine interferon-γ (IFN-γ) and interleukin 10 (IL-10) in *P.y*17XL-infected BALB/c mice on days 0, 3 and 5 p.i. after in vivo depletion of Tregs.

### IL-17 neutralization in vivo

Purified anti-IL-17A mAb (Biolegend, clone TC11-18H10.1) 100 mg was used to neutralize IL-17 in BALB/c mice by i.p. administration one day after *P.y*17XL challenge. Control group was given the same volume of PBS.by ip. The proportion of Th17 was detected by Flow cytometry.. Levels of cytokine IFN-γ and IL-10 were analyzed using spleen cell culture supernatants from *P.y*17XL-infected BALB/c mice on days 0, 3 and 5 p.i. after in vivo IL-17 neutralization.

### Detection of cytokines by ELISA

Mice were killed at the indicated time points (day 0, 3 and 5) and the splenocytes were havested in order to detect the levels of cytokines [16]. Concentration of spleen cells was adjusted to 10^7^ cells/ml using RPMI-1640 supplemented with 10% heat-inactivated FCS.. Aliquots (500 μl/well) of the cell suspensions were incubated in 24-well flat-bottom tissue culture plates (Falcon®) in triplicate for 48 h at 37 °C in a humidified 5% CO2 incubator. Following the supernatant fractions were collected and stored at − 80 °C until the detection of cytokines.

Commercial enzyme linked immunosorbent assay (ELISA) detected levels of IFN-γ and IL-10 in the supernatant samples (R&D Systems, Minneapolis, MN) according to the manufacturer’s instructions. The results were calculated by a standard curve generated using recombinant cytokines after obtaining the OD values.

### Cell surface staining, intracytoplasmic staining and flow cytometry

In order to analyze dynamic changes of splenic DCs (mDCs and pDCs), the expression of MHCII, CD80 and CD86 on CD11c^+^DCs, and the populations of Tregs and Th17 cells, BALB/c mice were sacrificed in the indicated time points. Antibodies were purchased from BD Biosciences.

Spleen cells from BALB/c mice were prepared in different time points after *P.y*17XL infections, and 1 × 10^6^ cells were stained. For mDCs and pDCs [17]: FITC-conjugated CD11c (clone HL3), PE-conjugated anti-CD11b (clone M1/70) and PerCP-conjugated anti-CD45R/B220 (clone RA3-6B2) were used. For mature DCs: FITC-conjugated anti-CD11c (clone HL3), APC-conjugated anti-MHC II (clone M5/114.15.2) (eBioscience), PerCP-conjugated anti-CD80 (clone 16-10A1) and PE-conjugated anti-CD86 (clone GL1) were used. For actived DCs, FITC-conjugated CD11c mAb (clone HL-3), PE-conjugated anti-IL-10 mAb (clone JES5-16E3) and PE-conjugated anti-IL-12 mAb (clone C15.6) were used to intracelllular staining for the expression of IL-10 and IL-12 in CD11c^+^DCs.

To assess Tregs [17], FITC-conjugated anti-CD4 (clone GK1.5) and PE-conjugated anti-CD25 antibodies (clone PC61) were added to 1 × 10^6^ spleen cells, which were resuspended in 100 μl of PBS supplemented with 1% FCS for surface staining. Then the cells were fixed and permeabilized, and intracytoplasmic staining was performed using APC-conjugated anti-Foxp3 (clone FJK16s, eBioscience) antibody.

To assess Th17 cells, FITC-conjugated anti-CD4 (clone GK1.5) and PE-conjugated anti-CD3 antibodies (clone 17A2) were added to 1 × 10^6^ spleen cells, which were resuspended in 100 μl of PBS supplemented with 1% FCS for surface staining. Then the cells were fixed and permeabilized [15], and intracytoplasmic staining was performed using APC-conjugated anti-IL-17A (clone TC11-18H10) antibody.

The cells were then washed twice with PBS containing 1% FCS and suspended in 300 μl of PBS. The cells were analyzed in a FACS Calibur cytofluorometer using CellQuest software.

### Statistical analysis

Data were presented as the mean value±standard error of the mean (SEM). Survival was assessed using the Kaplan–Meier (K–M) approach (SPSS 17.0). Statistical significance between two groups was determined using the Student’s t-test and between three or more groups by one-way ANOVA. *P*-values were calibrated using Bonferroni correction. The level of statistical significance was set at *p* < 0.05.

## Results

### The effectiveness of Tregs depletion in vivo

**Fig. 1:**
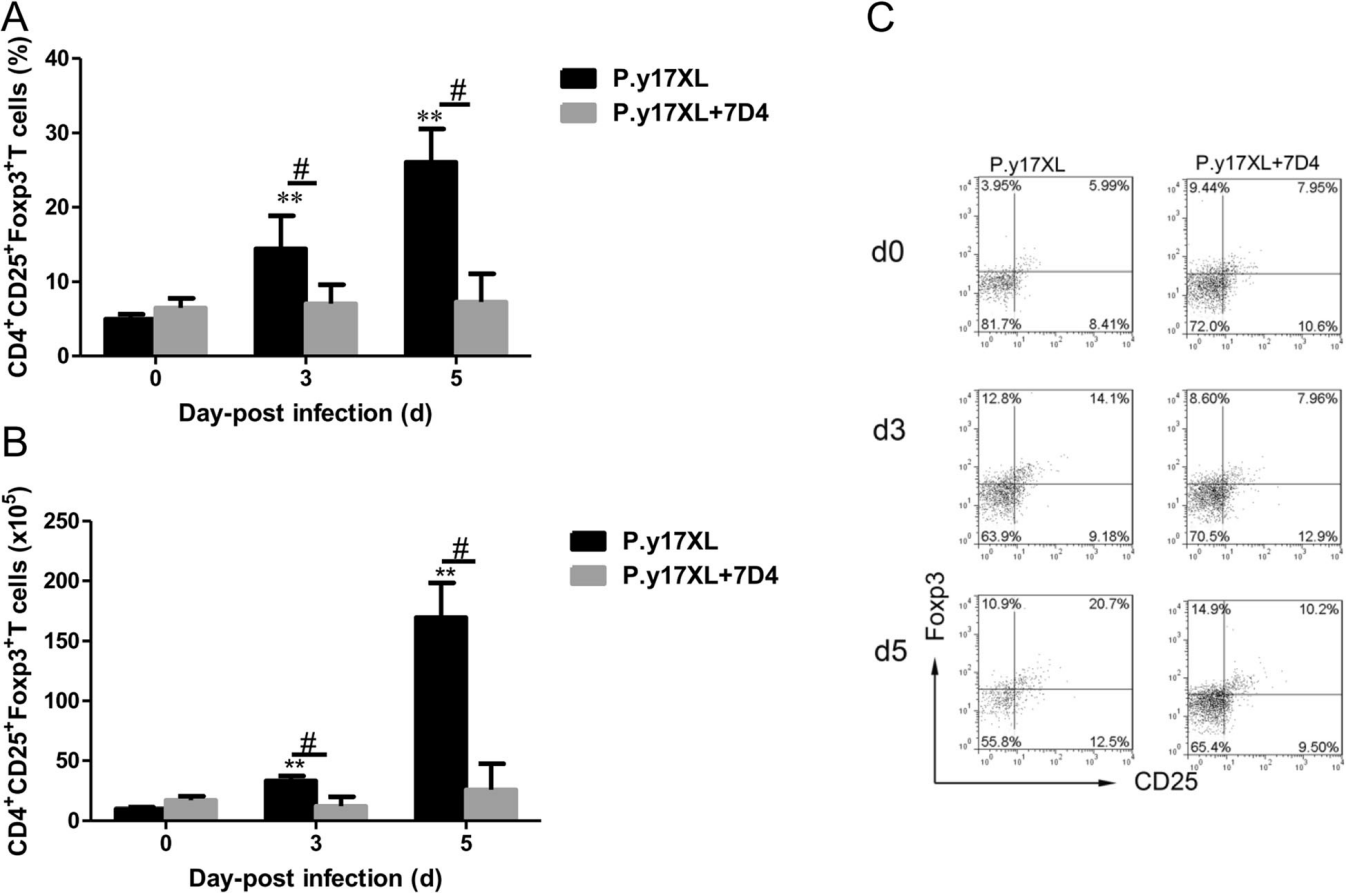
Flow cytometric analysis demonstrated the efficacies of Tregs depletion in vivo. The proportion of Tregs in CD4^+^ T cells (**a**), absolute cell numbers of Tregs (**b**), and dot plots (**c**) before and after anti-7D4 mAb treatment were detected. Column diagram (left) and representative dot plots (right) of Tregs in the infected with *P.y17XLP.y17XL* (normal infection) and CD25-depleted BALB/c *P.y17XL* (*P.y17XLP.y17XL* + 7D4) were displayed. Results are presented as arithmetic mean of 4 mice per group ± SE. Single asterisk (*) and double asterisks (**) indicates *P* < 0.05 and *P* < 0.01, respectively, compared with control mice (non-infected mice, 0d). Single pound sign (^#^) and double pound sign (^##^) indicates *P* < 0.05, and *P* < 0.01, respectively, between infected and Tregs depletion groups mice. * = Calculated value of ‘*p*’ by student’s t-test; # = Calculated value of ‘p’ by one-way ANON. *P*-values were calibrated using Bonferroni correction.The data are representative of three separate experiments

Flow cytometry was used to confirm the efficacy of Tregs depletion (Fig. 1). The proportion and absolute cell numbers of Tregs derived from CD25-depleted mice were significantly lower than that of control group with 78.5% reduction on day 3 pi and 83.5% reduction on day 5 pi.

### The effectiveness of Th17 neutralization in vivo

**Fig. 2:**
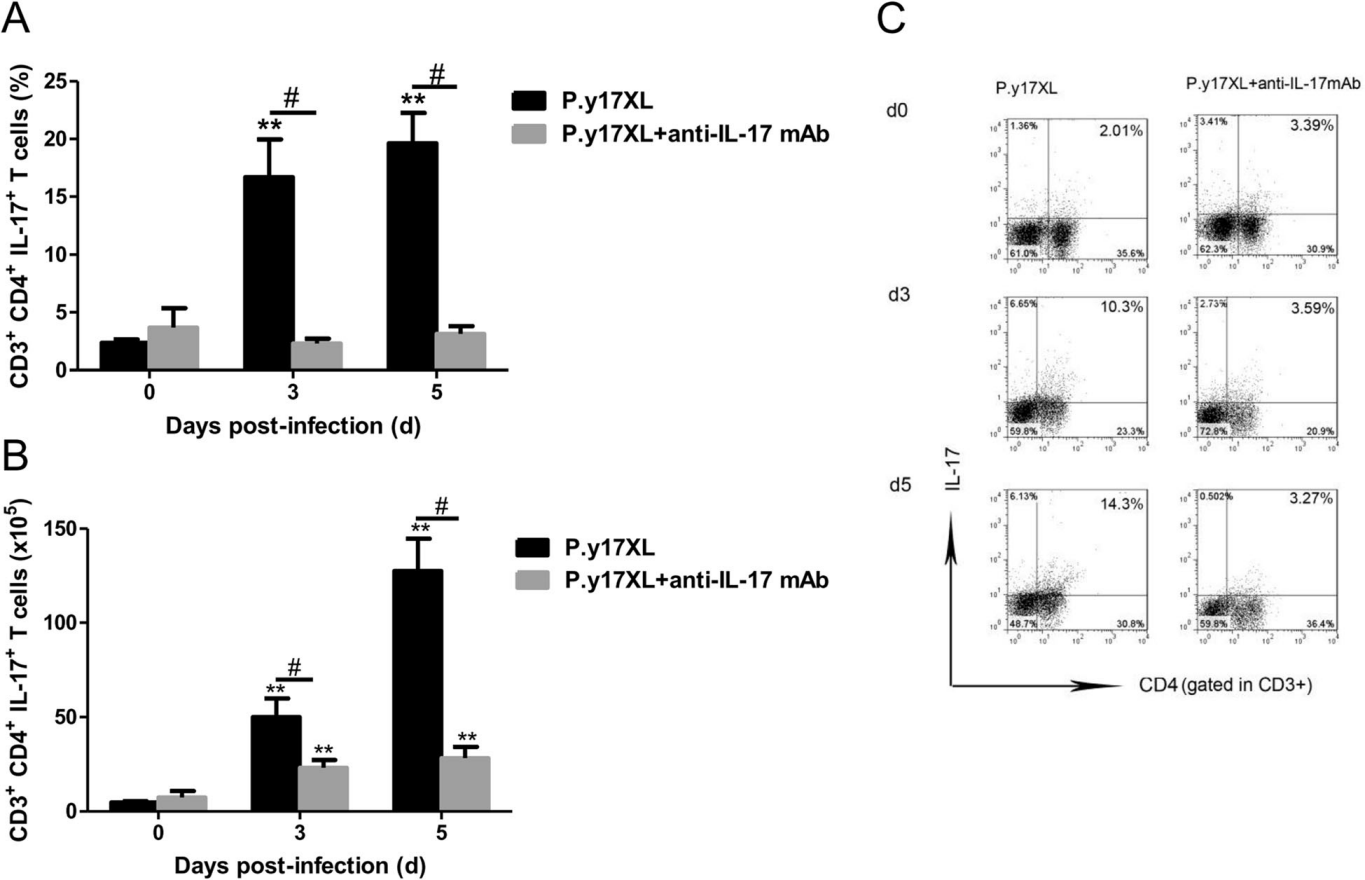
Flow cytometric analysis demonstrated the efficacies of Th17 neutrilization in vivo. The proportion of Th17 in CD4^+^ T cells (**a**), absolute cell numers of Th17 (**b**), and dot plots (**c**) after anti-IL-17 mAb treatment were detected. Column diagram (left) and representative dot plots (right) of Th17 in the infected with *P.y17XL* (normal infection) and CD25-depleted BALB/c *P.y17XL* (*P.y17XL* + anti-IL-17 mAb) were displayed. Results are presented as arithmetic mean of 4 mice per group ± SE. Single asterisk (*) and double asterisks (**) indicates *P* < 0.05 and *P* < 0.01, respectively, compared with control mice (non-infected mice, 0d). Single pound sign (^#^) and double pound sign (^##^) indicates *P* < 0.05, and *P* < 0.01, respectively, between infected and Tregs depletion groups mice.* = Calculated value of ‘*p*’ by student’s t-test; # = Calculated value of ‘*p*’ by one-way ANON. *P*-values were calibrated using Bonferroni correction. The data are representative of three separate experiments

Flow cytometry was used to confirm the efficacy of Th17 neutralization (Fig. 2). The proportion and absolute cell numbers of Th17 in CD4^+^ T cells derived from IL-17A mAb neutralization mice were as expected lower than that of control group with 86.2% reduction on day 3 pi and 84.1% reduction on day 5 pi.

### *P.y*17XL infection course in BALB/c mice treated with different factors

**Fig. 3:**
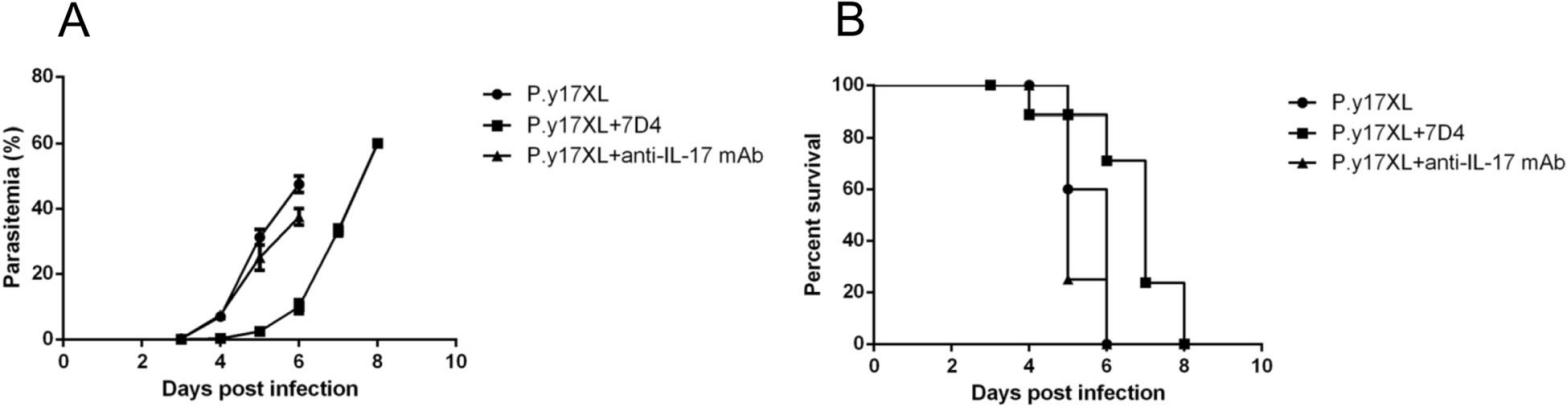
Parasitaemia (**a**) and survival rate (**b**) of *P.y*17XL infection in control and Tregs-depleted or Th17 neutralized BALB/c mice. Parasitaemia was calculated by counting the number of parasite-infected erythrocytes per 1000 erythrocytes. Mortality was monitored daily. The data were representative of three separate experiments

It is well known that different rodent models develop different outcomes when infected with the same strain of malaria parasite [15, 18]. In this study, BALB/c mice were infected with *P.y*17XL. As expected, *P.y*17XL-infected BALB/c mice developed a high parasitemia on day 6 p.i. with a peak around 45–50%, and all mice died. However, in Tregs depleted mice (7D4), parasitemia reached a peak around 60% on day 8 p.i., all mice died, but the lifetime was extended 2 days. Moreover, in Th17 neutralization mice, parasitemia merely reached 35–40% on day 6 p.i, but all mice died (Fig. 3a, b).

### Dynamics of pro- and anti-inflammatory cytokines in BALB/c mice infected with *P.y*17XL

**Fig. 4:**
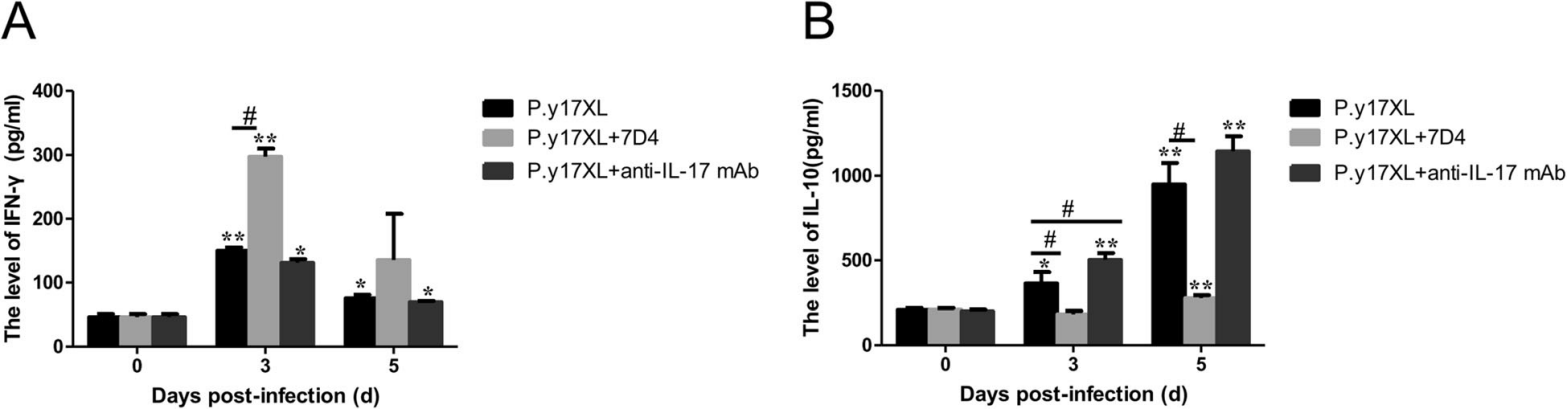
Effects of Tregs depletion or Th17 neutralization on the levels of pro- and anti-inflammatory cytokines during *P.y*17XL infection. **a** The level of IFN-γ production. **b** The level of IL-10 production. ELISA was performed on supernatants of cultured spleen cells from BALB/c mice. Results were presented as the arithmetic mean of 4 mice per group ± SE. Single asterisk (*) and double asterisks (**) indicated *P* < 0.05 and *P* < 0.01, respectively, compared with control mice (non-infected mice, 0d). Single pound sign (^#^) and double pound sign (^##^) indicates *P* < 0.05, and *P* < 0.01, respectively, between infected and Tregs depletion/Th17 neutralization groups mice. * = Calculated value of ‘*p*’ by student’s t-test; # = Calculated value of ‘*p*’ by one-way ANON. *P*-values were calibrated using Bonferroni correction.The data are representative of three separate experiments

To evaluate the relationship between inflammatory cytokines and the outcomes of BALB/c mice infected with *P.y*17XL, IFN-γ and IL-10 from the supernatants of cultured splenocytes with Tregs depletion or Th17 neutralization were assayed by ELISA. The pro-inflammatory cytokine IFN-γ in Tregs depletion mice appeared to be increased after infection with a peak 3 days (*P* < 0.05 and *P* < 0.01), and the concentration of IFN-γ was 2-fold as much as that in normal infection. IFN-γ in Th17 neutralization mice showed a slight decline on day 5 p.i. (Fig. 4a) during the course of *P.y*17XL infection. However, the anti-inflammatory cytokine IL-10 in Tregs depletion mice didn’t shown any significant changes compared with normal control mice on day 3 and 5 p.i.. In normal infection and Th17 neutralization mice, the levels of IL-10 were evaluated on day 3 and 5 p.i., which were 3–4-fold higher than those in Tregs depletion mice on day 5 p.i. (Fig. 4b).

### Analysis of the effects of Tregs depletion on DCs in BALB/c mice infected with *P.y*17XL

**Fig. 5:**
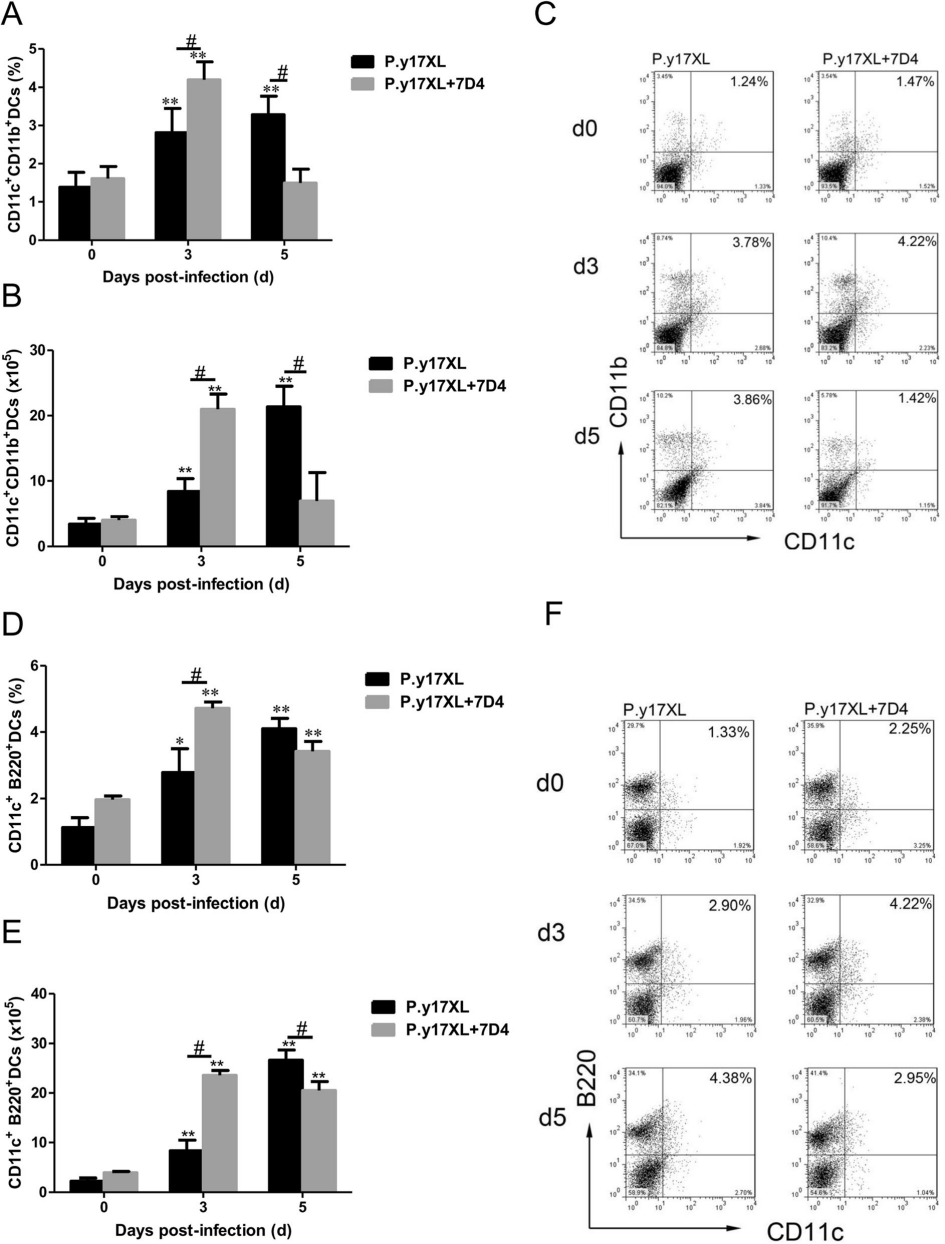
Effects of Tregs depletion on the subsets of DCs during *P.y*17XL infection. Proportion, absolute cell numbers (Column diagram, left) and representative dot plots (right) of mDCs (**a**, **b**, **c**) and pDCs (**d**, **e**, **f**) were displayed. Flow cytometric analysis was performed on spleen cells from BALB/c mice by double staining with FITC-anti-CD11c, PE-anti-CD11b and PerCP-anti-CD45R/B220. Results were presented as the arithmetic mean of 4 mice per group ± SE. Single asterisk (*) and double asterisks (**) indicated *P* < 0.05 and *P* < 0.01, respectively, compared with control mice (non-infected mice, 0d). Single pound sign (^#^) and double pound sign (^##^) indicates *P* < 0.05, and *P* < 0.01, respectively, between infected and Tregs depletion groups mice.* = Calculated value of ‘*p*’ by student’s t-test; # = Calculated value of ‘*p*’ by one-way ANON. *P*-values were calibrated using Bonferroni correction. The data are representative of three separate experiments

**Fig. 6:**
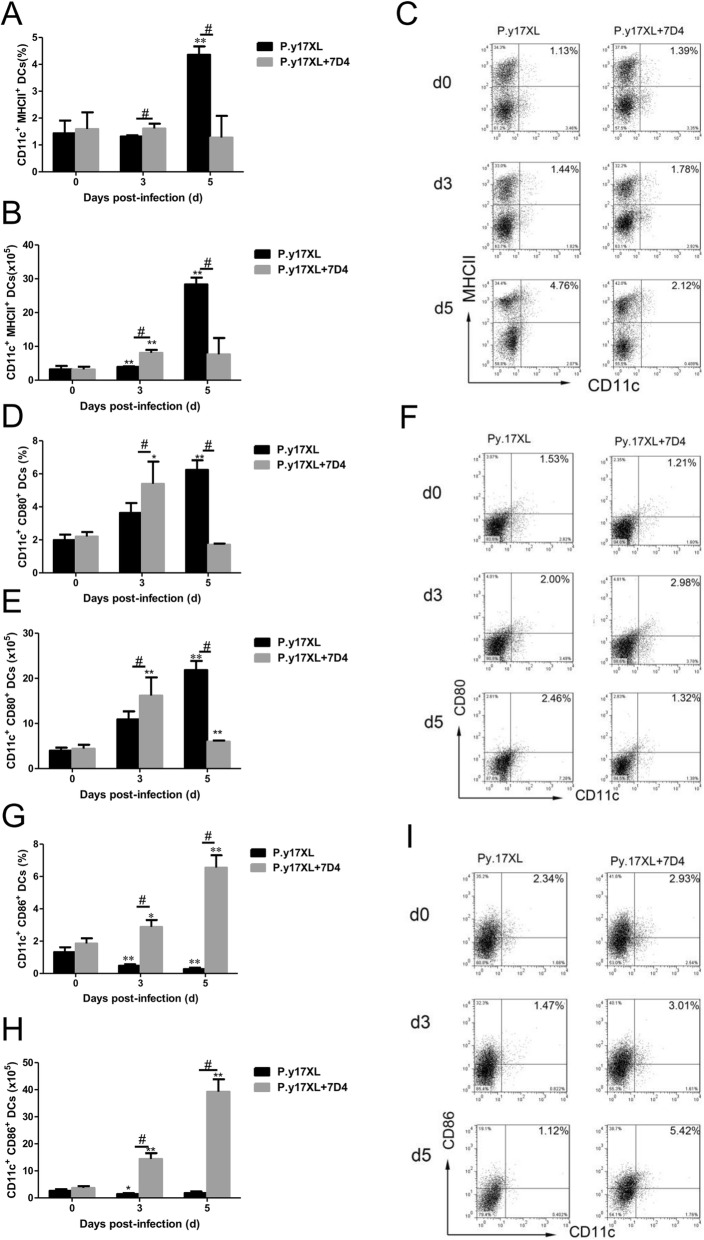
Effects of Tregs depletion on the surface molecular expression of DCs during *P.y*17XL infection. Proportion, absolute cell numbers (Column diagram, left) and representative dot plots (right) of MHC II (**a**, **b**, **c**), CD80 (**d**, **e**, **f**) and CD86 (**g**, **h**, **i**) on CD11c^+^ DCs were displayed. Flow cytometric analysis was performed on spleen cells from BALB/c mice by double staining with FITC-anti-CD11c, APC-anti-MHC-II, PerCP-anti-CD80 and PE-anti-CD86.Results were presented as the arithmetic mean of 4 mice per group ± SE. Single asterisk (*) and double asterisks (**) indicated *P* < 0.05 and *P* < 0.01, respectively, compared with control mice (non-infected mice, 0d). Single pound sign (^#^) and double pound sign (^##^) indicates *P* < 0.05, and *P* < 0.01, respectively, between infected and Tregs depletion groups mice.* = Calculated value of ‘*p*’ by student’s t-test; # = Calculated value of ‘*p*’ by one-way ANON. *P*-values were calibrated using Bonferroni correction. The data are representative of three separate experiments

**Fig. 7:**
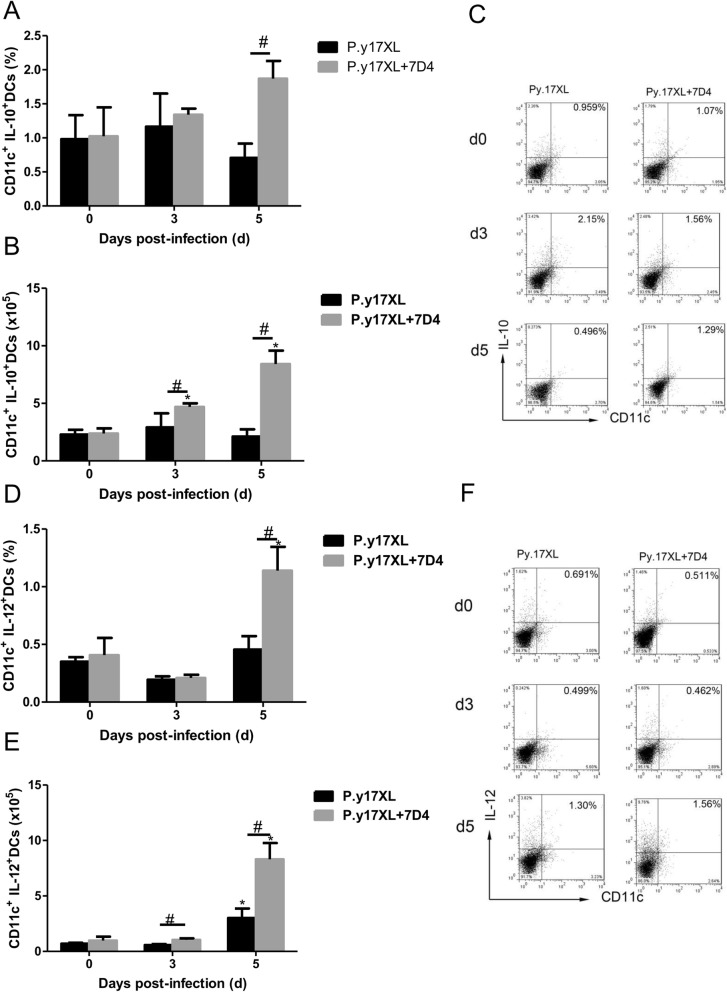
Effects of Tregs depletion on the dynamic of DCs-sectreting-cytokine during *P.y*17XL infection. Proportion, absolute cell numbers (Column diagram, left) and representative dot plots (right) of DCs-secreting-IL-10 (**a**, **b**, **c**) and DCs-secreting-IL-12 (**d**, **e**, **f**) on CD11c^+^ DCs were displayed. Flow cytometric analysis was performed on spleen cells from BALB/c mice by double staining with FITC-anti-CD11c, APC-anti-MHC-II, PerCP-anti-CD80 and PE-anti-CD86.Results were presented as the arithmetic mean of 4 mice per group ± SE. Single asterisk (*) and double asterisks (**) indicated *P* < 0.05 and *P* < 0.01, respectively, compared with control mice (non-infected mice, 0d). Single pound sign (^#^) and double pound sign (^##^) indicates *P* < 0.05, and *P* < 0.01, respectively, between infected and Tregs depletion groups mice. * = Calculated value of ‘*p*’ by student’s t-test; #=Calculated value of ‘*p*’ by one-way ANON. *P*-values were calibrated using Bonferroni correction. The data are representative of three separate experiments

In order to study if Tregs were involved in the regulation of DCs function and maturation in malaria, Tregs depletion was carried out using anti-CD25 mAb (7D4) before and after *P.y*17XL infection in BALB/c mice. We next examined the effects of Tregs depletion on regulation of DCs function and maturation. Both the percentages and absolute cell numbers of mDCs (Fig. 5a, b, c) and pDCs (Fig. 5d, e, f) increased and reached a peak in Tregs depletion mice on day 3 p.i. compared with normal infection group (*p* < 0.05), and then mDCs declined quickly to normal on day 5 p.i.; However, pDCs just gradually declined to normal infection levels. Moreover, we further compared the expression of maturation markers on DCs, Tregs depletion obviously enhanced the expressions of MHC-II, CD80 and CD86 (*p* < 0.05, Fig. 6a-i) on DCs on days 3 p.i.. Strikingly, the percentages of DCs-secreting IL-10 and IL-12 were significantly increased in Tregs depletion group on days 5 p.i. compared with normal infection group (*p* < 0.05, Fig. 7a-f).

### Analysis of the effects of Th17 neutralization on DCs in BALB/c mice infected with *P.y*17XL

**Fig. 8:**
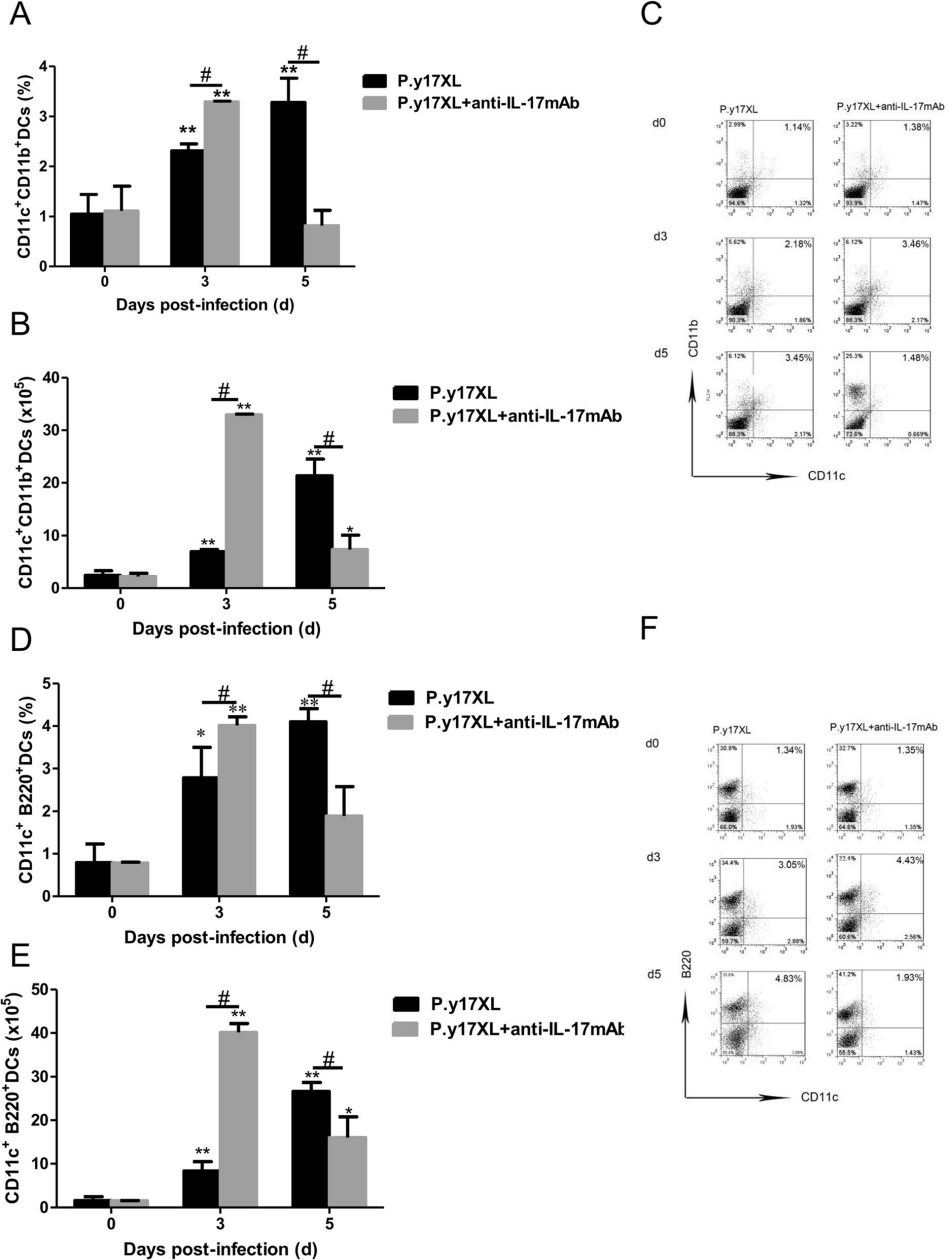
Effects of Th17 neutralization on the subsets of DCs during *P.y*17XL infection. Proportion, absolute cell numbers (Column diagram, left) and representative dot plots (right) of mDCs (**a**, **b**, **c**) and pDCs (**d**, **e**, **f**) were displayed. Flow cytometric analysis was performed on spleen cells from BALB/c mice by double staining with FITC-anti-CD11c, PE-anti-CD11b and PerCP-anti-CD45R/B220. Results were presented as the arithmetic mean of 4 mice per group ± SE. Single asterisk (*) and double asterisks (**) indicated *P* < 0.05 and *P* < 0.01, respectively, compared with control mice (non-infected mice, 0d). Single pound sign (^#^) and double pound sign (^##^) indicates *P* < 0.05, and *P* < 0.01, respectively, between infected and Tregs depletion groups mice.* = Calculated value of ‘*p*’ by student’s t-test; # = Calculated value of ‘*p*’ by one-way ANON. *P*-values were calibrated using Bonferroni correction. The data are representative of three separate experiments

**Fig. 9:**
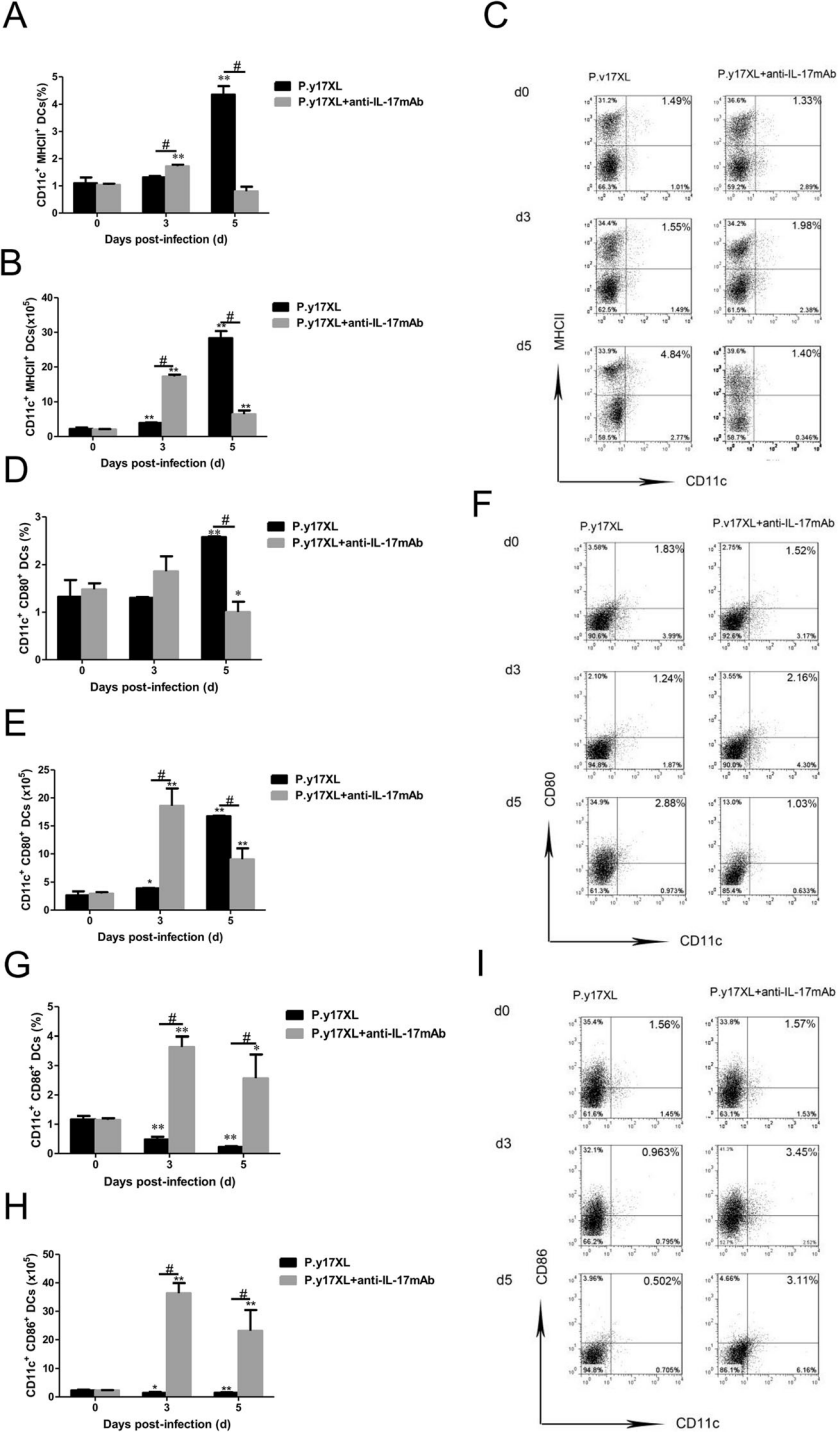
Effects of Th17 neutralization on the surface molecular expression of DCs during *P.y*17XL infection. Proportion, absolute cell numbers (Column diagram, left) and representative dot plots (right) of MHC II (**a**, **b**, **c**), CD80 (**d**, **e**, **f**) and CD86 (**g**, **h**, **i**) on CD11c^+^ DCs were displayed. Flow cytometric analysis was performed on spleen cells from BALB/c mice by double staining with FITC-anti-CD11c, APC-anti-MHC-II, PerCP-anti-CD80 and PE-anti-CD86.Results were presented as the arithmetic mean of 4 mice per group ± SE. Single asterisk (*) and double asterisks (**) indicated *P* < 0.05 and *P* < 0.01, respectively, compared with control mice (non-infected mice, 0d). Single pound sign (^#^) and double pound sign (^##^) indicates *P* < 0.05, and *P* < 0.01, respectively, between infected and Tregs depletion groups mice.* = Calculated value of ‘*p*’ by student’s t-test; # = Calculated value of ‘*p*’ by one-way ANON. *P*-values were calibrated using Bonferroni correction. The data are representative of three separate experiments

**Fig. 10:**
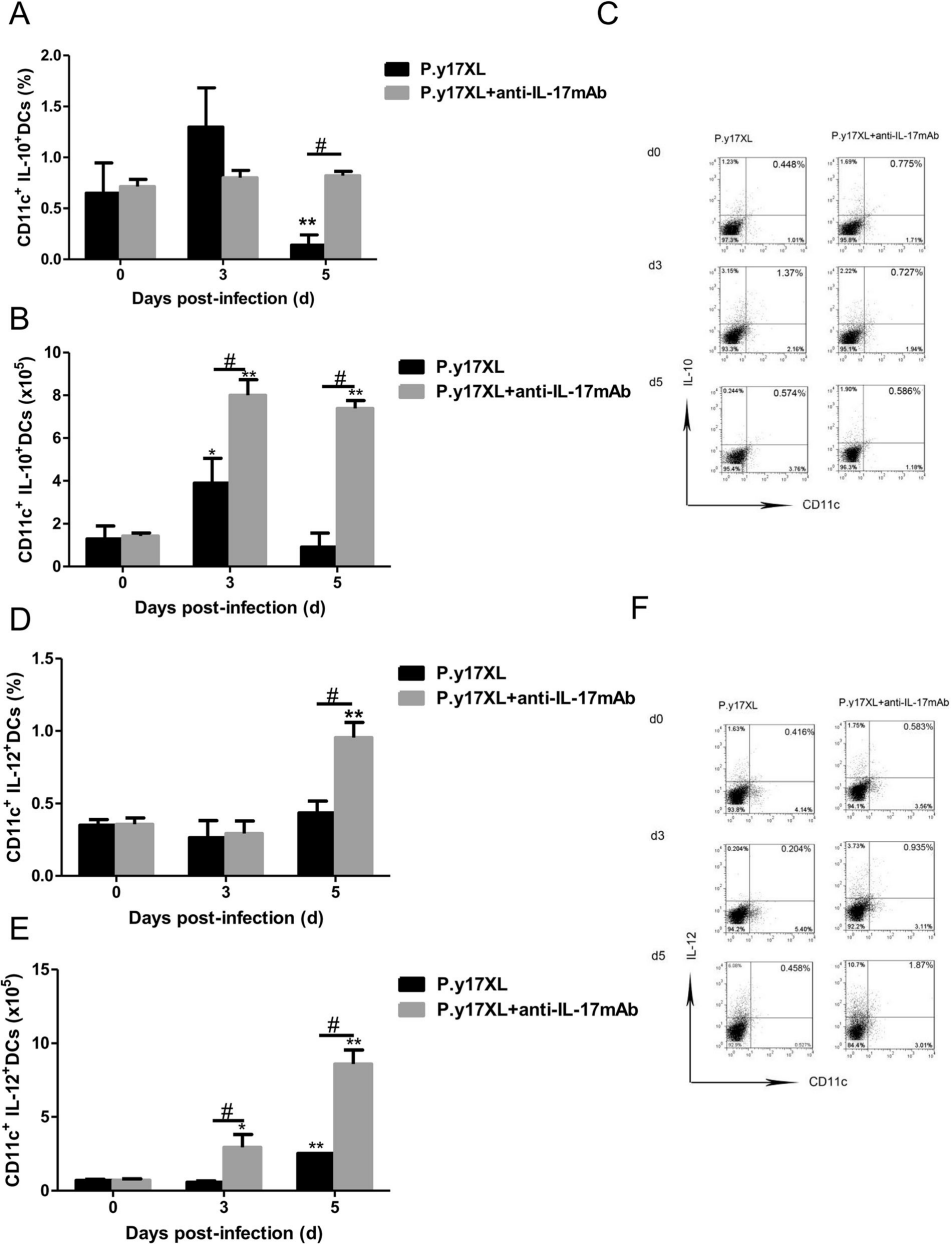
Effects of Th17 neutralization on the dynamic of DCs-sectreting-cytokine during *P.y*17XL infection. Proportion, absolute cell numbers (Column diagram, left) and representative dot plots (right) of DCs-secreting-IL-10 (**a**, **b**, **c**) and DCs-secreting-IL-12 (**g**, **h**, **i**) on CD11c^+^ DCs were displayed. Flow cytometric analysis was performed on spleen cells from BALB/c mice by double staining with FITC-anti-CD11c, APC-anti-MHC-II, PerCP-anti-CD80 and PE-anti-CD86.Results were presented as the arithmetic mean of 4 mice per group ± SE. Single asterisk (*) and double asterisks (**) indicated *P* < 0.05 and *P* < 0.01, respectively, compared with control mice (non-infected mice, 0d). Single pound sign (^#^) and double pound sign (^##^) indicates *P* < 0.05, and *P* < 0.01, respectively, between infected and Tregs depletion groups mice.* = Calculated value of ‘*p*’ by student’s t-test; # = Calculated value of ‘*p*’ by one-way ANON. *P*-values were calibrated using Bonferroni correction. The data are representative of three separate experiments

To ensure whether Th17 was also involved in the regulation of DCs function and maturation in malaria, Th17 neutralization was carried out using anti-IL-17 mAb post *P.y*17XL infection in BALB/c mice. We next examined the effects of Th17 neutralization on regulation of DCs function and maturation. Both the percentages and absolute cell numbers of mDCs (Fig. 8a, b, c) and pDCs (Fig. 8d ,e, f) increased in IL-17 neutralized mice on day 3 p.i. compared with normal infection group (*p* < 0.05), and then mDCs and pDCs declined quickly to normal on day 5 p.i. (*p* < 0.05). Moreover, we further compared the expressions of maturation markers, Th17 neutralization obviously enhanced MHC-II, CD80 and CD86 (*p* < 0.05, Fig. 9a-i) expression on DCs on days 3 p.i.. Strikingly, the percentages of DCs-secreting IL-10 and IL-12 were significantly increased in Th17 neutralization group on days 5 p.i. compared with normal infection group (*p* < 0.05, Fig. 10a-f).

## Discussions

CD4^+^ T helper cells are crucial for regulating appropriate immune response in inflammatory diseases. The existence of Tregs can actively suppress the function of conventional T cells, which is a key mechanism to limit inappropriate or excessive responses in the immune system [16]. However, it is very likely that Th17 cells contribute to inflammation and autoimmunity [16, 19]. Under specific conditions, CD4^+^ T cells can be induced to differentiate into these cell types and mediate immune responses by secreting different cytokines.. Relative studies revealed that Tregs, Th17 and Th1 are associated with early immune mechanisms by controlling *Plasmodium* infection [15, 20]. In addition, researchers clarified that antigen presentation and maturation of DC were impaired during *Plasmodium* infection, causing attenuation of specific humoral responses [10–13]. In this study, we showed that the intensity of Tregs and Th17 activation had an important role in regulating maturation and function of DCs during early *P.y*17XL infection, which is the key factor to the outcome of malaria infection.

Clinical symptoms of malaria infection were extremely complex, including severe anaemia, followed by acute respiratory distress, kidney failure, severe thrombocytopenia, coma, and shock syndromes [21]. It is generally agreed that T cell-mediated immunoregulation is consistent with the magnitude of effector response during malaria infections in both humans and mice [22]. In addition, innate immune response has been shown to limit parasite density [23], the key factors in eliminating blood-stage parasites are mainly antibodies (Abs) and T cells. From previous research, we have ensured that the effective establishment of Th1 immune response is beneficial for the resolution of the malaria infection [19, 24], and changes in number of Tregs were shown significantly to affect the final outcome of malaria infection [25]. Here, we further displayed that higher levels of IFN-γ and lower levels of IL-10 in Tregs depletion mice infected with *P.y*17XL on day 3 p.i. were noted (Fig. 4); Although all mice died, the lifetime was extended by 2 days compared to normal infection mice (Fig. 3), perhaps correlated with the disppearance of Tregs inhibition of Th1 response. Broadly speaking, Tregs depletion transmit a signal to T cells that the inhibition of Th1 response establishment was relieved briefly. When 7D4 was consumed away, the function of Tregs recovered rapidly and continue to trigger immunodepression. If we continue to treat *plasmodium* infected mice with 7D4 during the experiment, we may see an exciting results, which mortality is decreased in *plasmodium* infected mice, correlated with effective establishment of Th1 response. What we have to mention is that it is difficult to decide the treatment time points of 7D4, because we don’t know the shift time point from Th1 to Th2. This will be the main problem to solve in our future study. These data implied that Tregs, rather than Th17 take part in regulating the magnitude of *P.y*17XL-specific pro-inflammatory type 1 response in vivo. These observations also suggested that the regulation and induction of anti-malarial immune responses were important in determining the final outcome of infection.

In addition, there are two kinds of antibodies to deplete CD25^+^ cells: a combination of IgM (7D4) and IgG (PC61) antibodies leads to rapid and sustained abrogation of CD25 expression [26]. Kevin et al. observed that the 7D4 IgM antibody led to rapid but transient CD25 depletion with little or no effect on Foxp3+ cell numbers whereas PC61 was slower to act but eventually led to more complete and longer lasting depletion of both CD25+ and Foxp3+ cells. However, Kohm et al. who used 7D4 as their sole depleting Ab, showed that Foxp3+ cells was not depleted significantly, but there is a significant clinical effect. Thus they considered that anti-CD25 treatment leads to the functional inactivation of Tregs due to the loss of IL-2 signaling through CD25 [27]. Relative researches found that the maintenance of CD25^+^Foxp3^+^ cells required IL-2 assist critically in malaria. Though PC61 led to more complete and longer lasting depletion of both CD25^+^ and Foxp3^+^ cells, but it did not inactivate the functional Tregs. Following PC61 treatment, CD25 is rapidly re-expressed on peripheral CD25^−^Foxp3^+^ cells, the immunosupression function recover rapidly. In our previous studies, 7D4 was usually used to deplete Tregs in malaria [15], we found that the effectiveness of Tregs depletion by 7D4 is obvious, and is associated with recovery of DCs maturation and function. In fact, we used PC61 to deplete Tregs and observed the maturation and function of DCs in malaria. Although the effectiveness of Tregs by PC61 is incomparable, PC61 depletion had no effect on maturation and function of DCs. Thus we considered that Tregs depletion by PC61, just only a decreasion on number, not a function inactivation, and has not been used to clarify the real meaning of the experiment. Hence in this experiment, we chose 7D4 as the sole Ab to deplete Tregs.

DCs, as a professional APC, are highly specialized in presenting antigens to T cells [28], followed activating CD4^+^ T cells can fight against the parasites by producing inflammatory cytokines, leading to activation of other immune cells and Abs production of B cell [29]. In turn, cytokines from activated DCs stimulated differentiation of distinct CD4^+^ T lymphocyte profiles [30, 31]. IL-12 can induce generation of Th1 lymphocytes, which can control intracellular microorganisms [32–34]. IL-6 and TGF-β can induce generation of Th17, which can control extracellular bacteria and fungi [35], while TGF-β also induces generation of Tregs, which is important for controlling immune response [36]. However, we don’t found phagocytize and migrate of DCs towards to T cell-rich areas, correlated with immature DCs lose their abilities to initiate the adaptive immune response after taking on antigens [28]. Once DCs phagocytize iRBCs or free merozoites, it will verlieren an ability of parasites clearance [37]. Thus, the maturation and function of DCs were damaged in malaria, leading to subsequent failure of Th1 response establishment and the cytokines cascade reaction, following clear the *plasmodium* parasite in blood unsuccessfully. So far, we don’t know the reasons why the maturation of DCs is damaged. Thus, in this study we performed longitudinal assessments of the frequency, phenotype and function of circulating DCs in *P.y*17XL*-*infected BALB/c mice, especially after Tregs depletion or Th17 neutralization. We observed that the proportions of mDCs and pDCs were increased on day 3 p.i in Tregs-depleted or Th17-neutralized BALB/c mice, compared with normal infection group. Moreover, Tregs-depletion or Th17-neutralization also enhanced MHC-II, CD80 and CD86 expression on DCs on day 3 p.i.. Strikingly, the percentages of DCs-secreting IL-10 and IL-12 were significantly increased in Tregs-depletion or Th17 neutralization group on day 5 p.i. compared with normal infection group (Figs. 4, 5 and 6). Relative articles also demonstrated that Tregs can inhibit the protective cellular response by modifying DCs [38]. Similarly, we found that Tregs skewing inhibited the maturation and function of DCs during malaria infection. To be mentioned, Tregs and Th17 cells have the opposite effects on inflammation and immunologic tolerance, although they have the same T-cell precursors.. Th17, which obtains unique transcriptional profiles (STAT3, RORγ, and RORα), can produce IL-17, and required TGF-β, IL-6, IL-21, and IL-23 to help differentiation. Animals and humans experiments demonstrated that Th17/IL-17 take part in the fight against pathogens infection, including bacteria and viruses. The main reasons are correlate with Th17 regulating DC function, neutrophil recruitment, Th1 modulation, and Tregs balance [39]. Li et al. used the intracellular C. muridarum infected mice with IL-17 neutralization, and found that the function and activation of DCs were damaged, which the expression of CD40 and MHC II, IL-12 production were decreased, level of IL-10 was increased. Moreover, in vivo DCs isolated from IL-17-neutralized mice exhibit very poor ability to inhibit challenge infection compared with sham-treated mice, which display the effection of IL-17/Th17 on modulating DCs function to fight against Cm infection [40]. In contrast, our results found that the number, activation and function of DCs were significantly improved in IL-17-neutralized mice compared with normal infection group (Figs. 8, 9 and 10). Our results clarified that in vivo IL-17/Th17 amplification inhibited the maturation and function of DCs during malaria infection. Although we find the important significant of Th17 in malaria, their true clinical effect and regulatory mechanisms in anti-malaria immunity still remain many questions. In the next step, we will further explore the further molecular mechanisms of Th17 amplification on inhibiting maturation of DCs in malaria.

## Conclusions

Our study demonstrated that maturation and function of DCs are essential to malaria disease progression. It is closely regulated by the differentiation of Th17,and Tregs. *Plasmodium* infection in the early stage induces Tregs skewing and Th17 amplification by attenuating maturation and function of DCs. On the other hand, Tregs/Th17 inhibits the maturation and function of DCs by modifying the subsets of DCs, expression of surface molecules on DCs and secretion mode of cytokines.

## Supplementary information

**Additional file 1.** A summarizing statement in the methodology.

## Data Availability

Please contact the author for data requests.

## Abbreviations

*P.y17XL*: *Plasmodium yoelii* 17XL
Tregs: Regulatory T cells
DCs: Dendritic cells
APC: Antigen presenting cells
i.p.: intraperitoneal
PBS: Phosphate-buffered saline
FCS: Fetal calf serum
RT: Room temperature
ELISA: Enzyme linked immunosorbent assay
