# Supplementary material for: Plasmodium yoelii 17XL infection modified maturation and function of dendritic cells by skewing Tregs and amplificating Th17
Source: BMC Infect Dis. 2020 Apr 6;20:266. doi: 10.1186/s12879-020-04990-z (PMC7132900; doi:10.1186/s12879-020-04990-z)
Supplement: Supplementary file 1 — Additional file 1. A summarizing statement in the methodology. [file 12879_2020_4990_MOESM1_ESM.doc]

A summarizing statement in the methodology

In the current study, in Methods section, we used the methods from previously published works, please check the following references:

1. Methods of Mice, parasites, and experimental infection, Detection of cytokines by ELISA, Cell surface staining, intracytoplasmic staining and Flow cytometry refer to **GUANG CHEN**, JUN LIU, QING-HUI WANG, YI WU, HUI FENG, WEI ZHENG, SHENG-YU GUO, DONG-MEI LI, JI-CHUN WANG and YA-MING CAO. Effects of CD4+CD25+Foxp3+regulatory T cells on early Plasmodium yoelii 17XL infection in BALB/c mice. Parasitology. 2009, 136, 1107–1120.
2. Method of CD25 depletion refer to Jing-jing Wu, **Guang Chen**, Jun Liu, Tao Wang, Wei Zheng, Ya-Ming Cao. Natural regulatory T cells mediate the development of cerebral malaria by modifying the pro-inflammatory response. Parasitology International.2010; 59: 232–241
